# The next move in neuromodulation therapy: a question of timing

**DOI:** 10.3389/fncom.2014.00162

**Published:** 2015-02-13

**Authors:** Julien Modolo, Alexandre Legros, Anne Beuter

**Affiliations:** ^1^Human Threshold Research Group, Lawson Health Research InstituteLondon, ON, Canada; ^2^Departments of Medical Biophysics and Medical Imaging, Western UniversityLondon, ON, Canada; ^3^School of Kinesiology, Western UniversityLondon, ON, Canada; ^4^University of BordeauxBordeaux, France

**Keywords:** brain stimulation, Parkinson disease, closed loop control, human experimentation, computational neuroscience

## Introduction

The effect of time-varying electrical currents (AC) on neuronal activity is currently the focus of intense translational and multidisciplinary research efforts. Thanks to a broad range of neuromodulation modalities, it is indeed possible to induce, more or less invasively, electrical currents in brain tissue. On the non-invasive side, transcranial alternating stimulation (tACS), or transcranial magnetic stimulation (TMS) have demonstrated their potential for the symptomatic treatment of neurological disorders. In the domain of more invasive stimulation techniques, deep brain stimulation (DBS), or cortical stimulation (electrical motor cortex stimulation, EMCS) have proven extremely successful therapies for Parkinson's Disease (PD) or pain management, and are used in tens of thousands of patients worldwide (over 100,000 patients for DBS only). The underlying mechanisms are increasingly understood, even if they remain wrapped in some mystery that refrains the outstanding potential of brain stimulation for treating neurological disorders. However, the basic idea is simple: information processing by the brain is achieved, at least partially, by neuronal electrical oscillations in various frequency ranges, produced by a variety of neuronal networks distributed throughout the brain. Efforts to link the spatiotemporal structure of these neuronal oscillations with brain function and behavior have provided an enormous amount of data that is shaping our understanding of brain function (see Buzsáki and Draguhn, 2004 for a review on the functional significance of brain oscillations). By inducing currents in brain tissue, it is possible to modulate the membrane potential of neurons, thereby resulting in detectable changes in neuronal activity, and to impact associated function of neuronal networks.

The most widespread neurostimulation therapy today is DBS, clinically used to treat symptoms in neurological disorders such as in PD (see Modolo and Beuter, 2009 for a review). More than 25 years after its discovery, the technology of DBS has not changed much: high-frequency (>130 Hz) electrical stimulation using biphasic pulses, which are defined by their pulse width and amplitude. One minor recent innovation is the use of current-controlled DBS devices, which keep the stimulation steady at all times to avoid fluctuations in the stimulation signal being delivered and potential associated side effects (Bronstein et al., 2014). Given the tremendous progress of electronics over the last 25 years, and the advance in our qualitative and quantitative understanding of brain function, it is somewhat surprising that more personalized, sophisticated devices have not surfaced yet. Of course, using more advanced neuromodulation technologies would be meaningless if current technology was sufficient. With only 5–10% of patients eligible for DBS, a 1–3% rate of complications during surgery, batteries to replace every 4 years on average (under general anesthesia), stimulation parameters needing manual adjustments, and a complete absence of brain activity monitoring, there is however a consensus on the facts that current technology is not sufficient, and that the next generation of neuromodulation devices has to be pushed forward. Perhaps the most important aspect of all is the ability of novel neuromodulation devices to deliver stimuli with the right timing: with DBS, no matter what the ongoing brain activity is, the same stimulation pattern is continuously repeated. Why is that a limit, and why is it of fundamental importance to improve DBS drastically?

## Why does timing matter?

Increased quantitative understanding of brain oscillations, combined with knowledge from non-linear dynamics, has revealed numerous examples of non-linearities in brain activity. One example, related to DBS since it was hypothesized as a possible mechanism of action, is called “depolarization block”: when the stimulation frequency exceeds a certain threshold value, some neurons will stop increasing their firing frequency and will become silent (Beurrier et al., 2001). The underlying mechanism is fundamentally non-linear in nature, and counter-intuitive. Another example is the importance of the phase response curve of a system to incoming stimuli (for an overview, see Canavier, 2006): in the case of neurons, the neuronal response is sensitive to the stimulus phase. Therefore, in addition to “what” happens in neural networks, it is crucial to know “when” it does happen. In the area of neuromodulation, the idea of stimulation signals precisely timed to induce predetermined effects on neuronal activity is being actively explored, with “closed-loop” stimulation protocols (Modolo et al., 2010, 2011, 2012; Beuter et al., 2014).

An excellent experimental demonstration of the importance of timing was provided by Brittain et al. (2013). In this paper, the authors showed that an innovative electrical brain stimulation strategy was remarkably efficient to dampen pathological tremor in patients with PD. In brief, they found that a stimulation (delivered using tACS) applied to the motor cortex had maximal effects at tremor frequency (5 Hz), when a specific time delay was present between the tremor and the stimulation signals. This result is of crucial interest since it illustrates the role of timing, and also constitutes an experimental validation of a biophysical model that we have developed and published in 2010 (model of adaptive motor cortex stimulation in “closed-loop”—Modolo et al., 2010; and Patent application #EP09305432.8 by Beuter and Modolo, 2009). Our model indeed naturally predicts and explains the tremor reduction obtained by Brittain et al. (2013) using such a neuromodulation technique.

Specifically, the model derives equations providing the explicit form of a neuromodulation signal aiming to attenuate abnormal neuronal activity at a given frequency (Modolo et al., 2010). It was shown that, to be efficient, the stimulation signal needed to be at the same frequency as the targeted pathological rhythm, with a time delay dependent on neuroanatomical properties of cortical tissue (e.g., connectivity). The underlying mechanism in the model was related to the attenuation of neuronal activity at the pathological frequency (both for individual neurons and neuronal coupling). Because our model is biologically realistic, it successfully predicted that a tremor reduction would occur with neuromodulation as applied by Brittain et al. (2013), and offered a biologically plausible explanation supporting the observed clinical benefits. Furthermore, the model suggests ways to further attenuate PD symptoms, (tremor serving as a proxy) using this neuromodulation technique. Overall, the use of quantitative models appears inevitable to apprehend the complex, non-linear nature of brain activity. Furthermore, the possibilities of applications for closed-loop stimulation are not limited to PD, but are also considered for other neurological disorders, such as epilepsy (see for example Sun and Morrell, 2014 for a commercially available closed-loop stimulation system designed for epilepsy), and probably numerous others.

## Time to move on to novel developments in humans

Currently, most of the experimental research performed in the area of neuromodulation involves animals (e.g., mice, primates). The main motivation behind such animal research is to better understand the mechanisms by which therapeutic electrical stimulation of brain tissue can lead to a normalization of brain activity and an improvement of symptoms, and to test the efficacy of potential therapies. This approach seems reasonable, and is perfectly justified in certain neurological diseases such as epilepsy, where the involved pathological brain activity patterns and clinical manifestations of the disease are consistent between species. However, it should be kept in mind that over 80% of new drugs tested for efficacy and safety in mice fail when it comes to humans, which represents not only an enormous waste of resources, but also obviously of funding (Perrin, 2014). In the area of neuromodulation in PD, a more recent example illustrates that even using animals closer to humans than mice can be problematic. For instance, Drouot et al. (2004) showed clinical improvements in akinesia and bradykinesia following electrical stimulation of the motor cortex, along with a normalization of firing rates and synchronization in the STN and GPi. This significant finding was then followed by a clinical trial (http://clinicaltrials.gov/show/NCT00159172), which did not demonstrate similar improvements in humans and was stopped.

Another argument to encourage human research in neuromodulation is that, historically, the benefits of electrical stimulation of the brain have been discovered in humans (Bechtereva et al., 1975; Benabid et al., 1987) using neuromodulation hardware (e.g., electrodes) approved for use in humans. Since no major advancement of neuromodulation therapy for PD has been achieved using animal research, this should encourage human research instead. This is especially relevant since, nowadays, there are a multitude of devices approved for use in humans (flat electrodes, “Utah array,” DBS electrodes…), and using such devices to deliver stimuli that remain within limits of what is considered safe (e.g., in terms of frequency, amplitude, pulse width) should offer the possibility to test, in human patients, potential novel patterns of stimulation or “smart” stimulation devices delivering on-demand stimulation. The maturity of available neurostimulation hardware combined with the guidance of reliable biophysical models of brain tissue activity should convince researchers that now is the good timing to focus on human research regarding novel neuromodulation therapies in PD.

## Concluding remarks

The fact that biophysical models can predict the effects of electric stimulation of brain tissue on brain activity and associated symptoms reinforces the role of biophysical modeling as a driving force in engineering new neuromodulation therapies for PD. Biophysical modeling can therefore act at least as a tool to accelerate research and development, and might become in the future the preferred alternative to animal testing, given the growing success of the former and the increasingly recognized limitations of the latter. Such therapies could soon outperform open-loop DBS as it is done today. Let us also emphasize that human research should be supported to accelerate further clinical testing and validation of novel neuromodulation therapies, since the technology is available to make it happen safely. Finally, this convergence between biophysical modeling results and clinical results obtained in PD patients gives confidence that cheaper, on-demand, less invasive neuromodulation therapies with a well-identified mechanism of action will be available in the near future for these patients. Using mechanism-based approaches will represent a drastic shift in paradigm, ahead from empiric and evidence-based approaches that have been predominant to date.

### Conflict of interest statement

The authors declare that the research was conducted in the absence of any commercial or financial relationships that could be construed as a potential conflict of interest.
